# Different outcome of six homozygotes for prothrombin A20210A gene variant

**DOI:** 10.1186/1479-5876-6-36

**Published:** 2008-07-15

**Authors:** Pierpaolo Di Micco, Rosanna Di Fiore, Alferio Niglio, Sandro Quaranta, Antonella Angiolillo, Giuseppe Cardillo, Giuseppe Castaldo

**Affiliations:** 1 Department of Internal Medicine, Fatebenefratelli Hospital of Naples, Naples, Italy; 2 Department of Biochemistry and Medical Biotechnology, University of Naples "Federico II", Naples, Italy; 3 Department of Internal Medicine, Second University of Naples, Naples, Italy; 4 School of Sciences, University of Molise, Isernia, Italy; 5 CEINGE-Advanced Biotechnologies, scarl; University of Naples "Federico II", Naples, Italy

## Abstract

Prothrombin G20210A gene variant (FII G20210A) is a risk factor for venous thrombotic disease while conflicting results have been reported for the risk of arterial thrombotic events. However, vascular episodes were absent in up to 40% of the 67 homozygotes for the G20210A described so far, which indicates that the clinical expression depends on additional risk/trigger factors. We describe six homozygotes for the G20210A variant, among which the first pair of siblings (cases n. 3 and 4) reported so far that displayed a strongly heterogeneous clinical outcome. Case 1, a female of 27 years, developed a full thrombosis of common femoral, superficial and popliteal veins. She assumed oral contraceptives in the last two years. Case n. 2, 34 years old, suffered of recurrent pregnancy loss in absence of any causative alteration. Cases n. 3 and n. 5 experienced arterial thrombotic disease, i.e., juvenile myocardial infarction (40 years old) and stroke (48 years old), respectively, in absence of other risk factors. Finally, cases n. 4 and 6 identified as homozygotes for the FII G20210A variant being consanguineous of symptomatic subjects bearing the variant, did not experience any episode of venous nor arterial disease. Both of them have chronic liver disease with an impairement of the prothrombin time INR. Thus, homozygotes for the G20210A are at risk for arterial (in addition to venous) thromobotic events; chronic liver disease might modulate this risk.

## Background

The G20210A variant in the 3' UTR of prothrombin gene (FII G20201A) is associated with higher plasma prothrombin activity and has a prevalence of 1.2–4.6% in the general Caucasian population, and higher in patients affected by venous thromboembolism (VTE, [1,2]). Heterozygotes for the variant have an odds ratio for VTE ranging from 2 to 4 [3]. On the contrary, conflicting results have been reported for the risk of arterial thrombotic events in subjects bearing the G20210A variant [4-15]. The high incidence of the G20210A variant in the general population and a large-cohort meta-analysis suggest that heterozygosity for G20210A is a mild prothrombotic factor [10]. On the other hand, vascular episodes were absent in up to 40% of the 67 homozygotes for the G20210A described so far [3], which suggests that clinical expression in subjects bearing inherited thrombophilia appear only in the presence of additional risk factors [3,11]. The clinical history of six homozygotes for the FII G20210A variant studied by our group, among which the first pair of siblings described so far, indicates that the clinical outcome of these subjects is strongly heterogeneous, and that both trigger and protective factors may be involved.

## Case reports

### Case 1

female, current age 27 years. She takes oral contraceptives since the last two years. Few weeks ago color Doppler revealed a full thrombosis of common femoral, superficial and popliteal veins of right leg. Laboratory revealed she was homozygote for FII G20210A, with a normal activity of ATIII, PC and PS, the absence of the Factor V Leiden (FVL) and methylenetetrahydrofolate reductase (MTHFR) C677T variant and the absence of antiphospholipid syndrome. Moreover, the patient did not show further thrombotic risk factor as recent surgery, immobility, first degree relative affected by venous thromboembolism, malignancy nor risk factor for arterial thrombosis as smoking, hypertension, diabetes, dyslipidemia. After 4 months she is ongoing oral anticoagulation and discharged pill.

### Case 2

female, current age 34 years. She experienced 2 early pregnancy loss within the first 12 weeks and 1 late pregnancy loss at 22 weeks of gestation. Her personal and familial anamnesis were negative for previous thrombotic disorders. Any alteration that may explain recurrent pregnancy loss (i.e. uterine malformations, chromosomal alterations, endocrinological diseases, infective gynaecological or systemic diseases, chronic inflammatory diseases) was absent. She was homozygote for FII G20210A, without other laboratory alterations (i.e. PC, PS or ATIII deficiencies, FVL or MTHFR variants, anticardiolipin antibodies and lupus anticoagulant). Also this patient did not show further thrombotic risk factor as personal history of venous thromboembolism as recent surgery as recent immobility as first degree relative affected by venous thromboembolism as malignancy nor risk factor for arterial thrombosis as smoking as hypertension, diabetes, dyslipidemia. The patient actually does not take any type of antithrombotic treatment and of course she was invited to perform thromboprophylaxis in presence of further transient thrombotic risk factor.

### Cases 3 and 4

case 3 is a male, current age: 42 years. At the age of 40 years he had an acute myocardial infarction (AMI). His mother died at 36 years of age from AMI and his father died at 58 years of age from hepatocellular carcinoma on cirrhosis. He is a non-smoker and diabetes, dyslipidemia and hypertension were excluded as risk factors for venous thromboembolism as recent surgery, immobility and malignancy. The remote anamnesis was negative for venous and arterial thrombotic diseases. Laboratory showed normal activity of ATIII, PC and PS, the absence of the FVL and heterozygosis for the MTHFR C677T variant with normal serum levels of homocysteine (Hcy). He was homozygote for FII G20210A variant. After the screening for thrombophilia his antithrombotic treatment was changed and actually based on the association of oral anticoagulation, with International Normalized Ratio, (INR, [12]), ranged between 2.0 and 3.0, and low doses of aspirin (i.e. 100 mg daily). Further, clinical, instrumental and laboratory findings excluded any liver disease. After genetic counselling, molecular analysis was extended to the family, and the patient's brother, case n. 4, current age 48 years old, was found to be homozygote for FII G20210A too. Anamnesis, clinical examination and instrumental tests (i.e. electrocardiography, echocardiography and vascular ultrasound scan of the carotid and lower limbs) excluded any vascular disease. Moreover, besides familial trend to thrombotic events and presence of inherited thrombophilia, as for his brother, further thrombotic risk factors for venous and\or arterial thrombosis were absent (see above). From the age of 21 years, he assumed high amounts of alcohol and developed liver cirrhosis, currently Child-Pugh stage B [16], with a prothrombin INR of 1.9. None antithrombotic treatment was suggested for him.

### Case 5

female, current age: 60 years old. Both parents died when she was a child, the mother for AMI, the father for an unknown disease. Remote anamnesis was negative for thrombotic diseases and for atherothrombosit risk factors as smoking, alcohol, diabetes, dyslipidemia, hypertension and pharmacological and hormonal therapies. Similarly, risk factors for venous thrombosis as recent immobility, malignancy and recent surgery were absent. At the age of 48 years, she experienced an episode of left hemiparesis. Computerized tomography scan showed hypodensity in the talamus and in the motor cortical area. Carotidal and vertebral vascular ultrasound scan associated to color-Doppler, and ECG and echocardiography were negative. The patient was successfully treated with antithrombotic therapy. Laboratory demonstrated homozygosity for the FII G20210A gene variant, whereas other data (i.e., AT III, PC, PS, homocysteinemia, FVL and MTHFR variants) were normal. The antithrombotic therapy based on oral anticoagulation (INR range 2.0–3.0) is ongoing again and 12 years later (today), the patient did not refer any other thrombotic episode.

### Case 6

male, current age: 40 years. This patient came to our attention because his sister, who suffered from recurrent abortions, was heterozygous for the FII G20210A variant. After genetic counselling, the analysis was extended to all first-degree relatives and he resulted to be homozygous for G20210A. All other laboratory data (i.e., AT III, PC, PS, FVL and MTHFR C677T variant) were normal. His father died at 40 years from AMI; his mother (heterozygote for FII G20210A) had never suffered from vascular diseases. The patient is a non-smoker, without hypertension, diabetes and\or dyslipidaemia; thrombotic risk factors for venous thromboembolism were also absent as recent surgery as recent immobility as malignancy and its therapies as previous personal history of venous thrombosis. Remote and recent anamnesis are negative for vascular diseases and these data were confirmed by a clinical examination and additional tests (i.e. electrocardiography, echocardiography and vascular ultrasound scan of carotid and lower limbs). The patient has a 20-year history of intravenous drug assumption (mainly heroin) and is affected by chronic viral hepatitis C and Child-Pugh stage A cirrhosis with a INR < 1.7 [16]. No episode of vascular disease occurred during the last 4 years and none antithrombotic treatment was suggested for him.

## Discussion

Two of the six described cases homozygotes for the G20210A prothrombin variant experienced a deep vein thrombosis during oral contraceptive use (one of them) and recurrent pregnancy loss (the other one). Two other had an acute thrombotic arterial event. The last two, bearing the same genotype, did not experience any thrombotic arterial nor venous disease, and both were affected by chronic liver disease with an altered prothrombin activity. The first two cases confirm the well known association between inherited thrombophilia and venous thromboembolism and\or recurrent pregnancy loss [17,18]. Case n. 1 was a young woman and had a deep vein thrombosis of lower limb after two years of oral contraceptive use. Indeed, several studies described the early onset of venous thromboembolism in subjects bearing inherited thrombophilia [17] and the trigger action of oral contraceptives in particular in subjects with already known thrombophilia [19]. Similarly, the relationships between inherited thrombophilia and pregnancy loss are well known [18]. Our patient experienced three consecutive pregnancy losses without any known clinical, anatomic or genetic alterations causing adverse pregnancy outcomes other than the homozygosity for FII G20210A variant. Interestingly, the patient suffered of two episodes of early recurrent pregnancy losses and one episode of late pregnancy loss that are more frequently associated to inherited thrombophilia [18]. Furthermore, our cases support that the homozygosis for the FII G20210A variant may be a risk factor also for arterial thrombotic events besides of venous thromboembolism. Both the patients who experienced arterial thrombotic diseases had a parent who died from AMI and thrombotic episodes occurred at a relatively young age (40 and 48 years, respectively). Furtherly, they did not have other common risk factors for thrombotic arterial disease such as smoking, hypertension, diabetes, dyslipidemia [20]. Conflicting data have been reported so far on the relationships between inherited thrombophilia and arterial thrombotic diseases [4-15]. A higher risk of AMI in young women carrying FII G20210A variant and an association between G20210A and coronary heart disease were reported. Other retrospective and prospective studies excluded that the G20210A variant may predispose to arterial thrombotic diseases. G20210A was not associated to an increased risk of infarction or stroke in a prospective study of 15,000 males [15], while a meta-analysis involving 66,000 cases and 91,000 controls indicated that the G20210A variant was associated to a moderately increased risk of coronary diseases [10].

Finally, other two described cases suggest that chronic liver disease may mitigate the risk of vascular disease in patients bearing the G20210A genotype. This information may be found as an innovative point of view on the gene-enviroment relationships existing in the pathogenesis of thrombotic diseases. In fact, patients 4 and 6, who did not have a history of vascular diseases, had liver disease (i.e., Child B and Child A cirrhosis in cases 4 and 6 respectively). Liver cirrhosis is typically associated to impaired synthesis [21] and post-translational maturation [22,23] of clotting factors, which increases the trend to develop hemorrhagic complications as acute gastrointestinal bleedings [24]. Consequently, these biochemical alterations may counteract the increase of prothrombin levels and activity that are associated to the G20210A variant [25]. Indeed, patients 4 and 6 had reduced INR according to the hypocoagulability due to the impaired liver function. In this setting it must be underlined that patients 5 and 6 were identified as homozygotes for FII G20210A being consanguineous of other affected subjects, and this evidence reinforces the role of the genetic counselling to the families, and the concept to extend molecular analysis to consanguineous once a symptomatic subject bearing inherited thrombophilia is identified. In conclusion, our cases support the idea that the G20210A homozygote genotype associated to another thrombotic risk factor acting as trigger factor is sufficient to give vascular complications as venous thromboembolism and\or female infertility [17,18]; moreover, inherited thrombophilia and in particular the prothrombin A20210G variant is a relevant risk factor for recurrent pregnancy loss. On the other hand prothrombin A20210G may be also a risk factor for arterial disease, as AMI, in particular if early onset and familial anamnesis were positive also in absence of further risk factors for arterial disease. Furthermore, although data need to be confirmed on a larger population, we suggest also that concomitant hypocoagulation, due to impaired liver function, may reduce the risk of venous and arterial thrombotic disease in homozygotes for the G20210A prothrombin variant.

## Conclusion

So, due to the lacking data available in the Literature, our case series underlined that the natural history of subjects carrying A20210G variant of prothrombin may be difficult to be evaluated in such situation. Relevant clinical aspect that should be considered are related to the strong association with repeated pregnancy loss and venous thromboembolism, in particular if further risk factor is present. On the other hand, other clinical aspects that should be considered also by further studies are the possible association with arterial thrombosis in presence\absence of further risk factor for atherothrombosis and the role of comorbidities with a trend toward hypocoagulation that may mitigate thrombotic trend as liver dysfunction in our cases.

## Competing interests

The authors declare that they have no competing interests, nor any financial supports during patients' selection, experimental tests and article extension.

## Authors' contributions

PDM and AN performed patients' selection. RDF and SQ performed all biochemical tests described in the paper. GCard and AA performed revision of the Literature. PDM and GCas performed paper extension.
